# Dry Eye Syndrome in Patients with Diabetes Mellitus: Prevalence, Etiology, and Clinical Characteristics

**DOI:** 10.1155/2016/8201053

**Published:** 2016-04-26

**Authors:** Xinyuan Zhang, Lin Zhao, Shijing Deng, Xuguang Sun, Ningli Wang

**Affiliations:** Beijing Institute of Ophthalmology, Beijing Tongren Eye Center, Tongren Hospital, Capital Medical University, Beijing 100730, China

## Abstract

There has been substantial progress in our understanding of the ocular surface system/lacrimal function unit in the past 15 years. Keratoconjunctivitis sicca, more commonly referred to as dry eye syndrome (DES), is the most frequently encountered condition and diabetes mellitus (DM) has been identified as one of the leading causes of DES. Poor glycemic control affects both the anterior and the posterior segments of the eye and increasing prevalence of diabetes-associated DES (DMDES) has been reported in recent years. The pathogenesis and specific features of DMDES remain uncertain and interventions are limited to those used in DES. This review outlines the pathogenesis, clinical manifestations, and the current preventive and treatment strategies for diabetes-related DES.

## 1. Introduction

The International Diabetes Federation (IDF) estimates that the global diabetes epidemic continues increasing. According to the report of the IDF in 2013, China has the largest number of diabetics (98.4 million) and this number is now higher than in India (65.1 million) and in the USA (24.4 million) [1].

While diabetic retinopathy (DR) and diabetic cataracts are well-known complications, dry eye syndrome (DES), also referred to as keratoconjunctivitis sicca, is also common in the diabetic population. Studies have indicated 54% prevalence of asymptomatic and symptomatic DES, in diabetes [2]. However, the relationship between diabetes and DES still remains unclear. This review aims to discuss the prevalence, etiology, and treatment strategies of diabetes mellitus associated DES and to emphasize the importance of early diagnosis and interventions in diabetes-associated DES.

## 2. Prevalence of Dry Eye Syndrome in Diabetes Mellitus

Diabetes mellitus (DM) has been identified as one of the leading systemic risk factors for DES. The reported prevalence of DES in diabetics is 15–33% in those over 65 years of age and increases with age and is 50% more common in women than in men [3]. The incidence of dry eye is correlated with the level of glycated hemoglobin: the higher the level of glycated hemoglobin, the higher the incidence of dry eye [4].

The Beaver Dam Eye Study reported that approximately 20% of dry eyes occurred in individuals with Type 2 diabetes aged between 43 and 86 years. Hom and De Land reported that 53% of patients with either diabetes or borderline diabetes had self-reported, clinically relevant dry eyes [5]. In a hospital-based study, 54% of those with diabetes had DES and there was a significant correlation between DES and the duration of diabetes. This suggests that examination for dry eye should be an integral part of the ocular examination in patients with diabetes [2].

Significant associations have been identified between diabetic retinopathy (DR) and DES. In a hospital-based study, 17.1% of DES in patients with DM was found to have mild nonproliferative diabetic retinopathy (NPDR), 17.1% had moderate NPDR, 11.1% had severe nonproliferative diabetic retinopathy (NPDR), and 25.1% had proliferative diabetic retinopathy (PDR) [6]. DR is also associated with a decrease in tear film function. Tear break-up time (BUT) and Schirmer's test values were significantly decreased in the PDR group compared to the non-DR group while corneal fluorescein staining scores, positive rate of rose Bengal staining, the surface regularity index, and the surface asymmetry index were increased. The concentrations of lactoferrin and tear-specific prealbumin were decreased in the DR group [6]. Another hospital-based study showed that DES is more prevalent in individuals with DR and/or clinically significant macular edema (*P* = 0.006) compared to the non-DR group. The odds of DR in DES were 2.29 (CI = 1.16–4.52, *P* = 0.016) and both DES and retinopathy were associated with HbA1c [7].

## 3. Classification of Dry Eye Syndrome

DES was recognized as a lacrimal function unit (LFU) dysfunction disease by the International Dry Eye Workshop in 2007. The LFU which protects and maintains the tear film and normal function of the ocular surface is composed of “the cornea, conjunctiva, lacrimal gland, meibomian gland, lids, and the sensory and motor nerves that connect them” [8]. Human tear film comprises three layers: lipid (secreted by the meibomian gland), aqueous (secreted by the lacrimal gland), and mucin (secreted by conjunctiva, cornea, lacrimal gland, and other structures). These three layers contain enzymes, signaling molecules, and metabolites and are essential in maintaining the physiological function of the ocular surface [9].

The 1995 NEI/Industry Dry Eye Workshop identified two types of DES: aqueous tear-deficient (tear-deficient, lacrimal tear deficiency) and evaporative dry eye. Aqueous-deficient dry eye has two major subgroups: Sjögren and non-Sjögren syndrome. Evaporative dry eye may be intrinsic (e.g., due to meibomian gland dysfunction, eyelid problems, or low blink rate) or extrinsic (e.g., due to vitamin A deficiency, preservatives in topical medications, contact lens wear, or diseases of the ocular surface) [10]. DM associated dry eye may be tear-deficient or evaporative dry eye [7].

## 4. Etiology of Diabetes Mellitus Associated Dry Eye Syndrome

LFU plays a regulatory role in tear secretion and tear film formation and maintains the normal physiology of the ocular surface; damage to any component of LFU leads to tear-deficient or evaporative DES.

**Figure 1:**
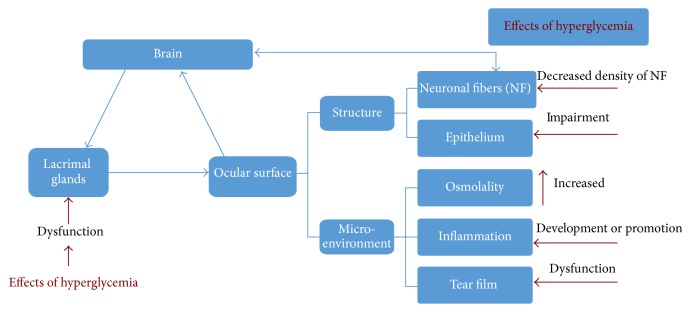
Lacrimal function unit (LFU) is composed of the “cornea, conjunctiva, lacrimal gland, meibomian gland, lids, and the sensory and motor nerves that connect them,” which protect and maintain the tear film and normal function of the ocular surface. LFU plays a regulatory role in tear secretion and tear film formation to maintain the normal physiology of the ocular surface; damage to any component of LFU leads to tear-deficient or evaporative diabetes mellitus associated dry eye syndrome.

Tear hyperosmolarity and tear film instability caused by LFU and ocular surface dysfunction are the key factors in DES. Effects of hyperglycemia on any component of the LFU may be transferred to the entire system via neural connections, leading to insufficient tear production or excess tear loss, abnormalities in blinking, and changes in tear film composition [10]; all these cause DES. The feedback loop for tear secretion and impact of diabetes mellitus on ocular surface and tear production are summarized in Figure 1.

### 4.1. Lacrimal Functional Unit Dysfunction

Patients with Type 1 or Type 2 Diabetes are at increased risk of developing LFU dysfunction [11].

DM is a risk factor for corneal epithelial abnormalities. DM causes epithelial barrier dysfunction which subsequently leads to corneal complications and then LFU dysfunction [11]. Diabetes with increased serum HbA1c levels is more predisposed to impaired barrier function in the corneal epithelium [11]. In a diabetic rabbit corneal epithelium dysfunction model, increased levels of glucose, glycogen, and sorbitol have been identified in the diabetic corneal epithelium as compared to controls suggesting that sorbitol pathway activation is involved [12].

The corneal complications caused by hyperglycemia include superficial punctate keratopathy, trophic ulcers, persistent epithelial defects, and recurrent corneal erosions; all these associated with DES [13]. It has also been shown that diabetics have lower values of tear secretion and tear break-up time test (TBUT).

In aC57BL/6Jdb/db mice model of DMDES, tear production substantially decreased concomitantly with a wounded corneal epithelium. Oxidative stress in the cornea was significantly increased with decreased SIRT1 expression [14]. The mean conjunctival staining scores were significantly increased in a diabetic group (*P* = 0.034) compared with a nondiabetic group [15]. The development of DES in association with antigen-specific insulitis and diabetes in a diabetes mice model has also been demonstrated [16].

### 4.2. Abnormal Tear Dynamics

#### 4.2.1. Abnormal Enzyme Metabolism

Aldose reductase is an important enzyme in the pathway involved in the pathogenesis of dry eye and oral administration of aldose reductase inhibitors has been demonstrated to improve tear dynamics [17, 18]. The polyol pathway is triggered by high glucose in Type 2 diabetes, inducing the activation of aldose reductase. It has been shown that the accumulation of sorbitol within cells leads to cellular edema and dysfunction, which ultimately results in lacrimal gland structure damage and dysfunction and the induction of decreased tear secretion.

#### 4.2.2. Decreased Mucin Secretion

In humans, mucosal and ocular surfaces are covered and protected by a high-molecular weight, heavily glycosylated protein, which is secreted by goblet cells and exogenous glands. About 20 basic types of mucins have been identified throughout the human body; at least 7 or 8 types of mucins are found in ocular surface. Tear mucin is secreted by the conjunctival goblet cells and conjunctival and corneal epithelial cells and contributes to the mucus layer. In addition to its protective effect, mucin also forms the glycocalyx that contributes to cell adhesion and makes the tear film hydrophilic. Diabetes causes corneal and conjunctival epithelial damage, inducing reduction of the number of goblet cells; it reduces mucin production and the hydrophilic nature of the ocular surface leading to tear film instability [2, 18].

### 4.3. Diabetic Neuropathy

Diabetic neuropathy may be an important risk factor for lacrimal gland dysfunction. Nakata et al. demonstrated that diabetes suppresses hemodialysis-induced increases in tear fluid secretion, which suggests that autonomic control of lacrimal gland function may be compromised by neuropathy in patients with DM [19].

Nerve fibers play an important role in the maintenance of normal function of the cornea and the integrity of the LFU. Hyperglycemia causes corneal epithelium barrier dysfunction and corneal neuropathy, subsequently triggering the trophic effects of the cornea dysfunction [20]. Chronic sensorimotor distal symmetric polyneuropathy (PN) is the most common form of diabetic neuropathy and is characterized by sensory and motor deficits. DES is particularly common in patients with Type 2 diabetes complicated with polyneuropathy (PN) [21]. Impaired corneal neurons and reduced corneal sensitivity have been reported in diabetic patients with PN [21]. Myelinated A-*δ* and unmyelinated C fibers are the main neural components of the human cornea. There is a significant difference in DES between those with diabetic PN, those without diabetic PN, and control subjects. The values of Schirmer's *I* test, TBUT, and corneal sensitivity were also worse in patients with PN compared to diabetics without PN and normal controls (*P* < 0.001) [18]. These findings suggest that patients with PN should be considered for testing for DES to prevent ocular surface impairment during the follow-up.

### 4.4. Tear Film Dysfunction

The tear film is the most dynamic structure of the LFU. It plays an important role in regulating epithelium function and interacting with surrounding tissues [22]. Tear film dysfunction has been found to be closely associated with DES. Chronic tear secretion deficiency and tear film dysfunction have also been identified in patients with diabetes [23, 24]. The tear lipid thickness (especially the lipid layer of the tear film), stability, corneal sensitivity, and tear quantity were significantly decreased in patients with diabetes. Tear film stability was inversely associated with the total neuropathy score [25].

## 5. Pathogenesis of DM Associated Dry Eye Syndrome

**Figure 2:**
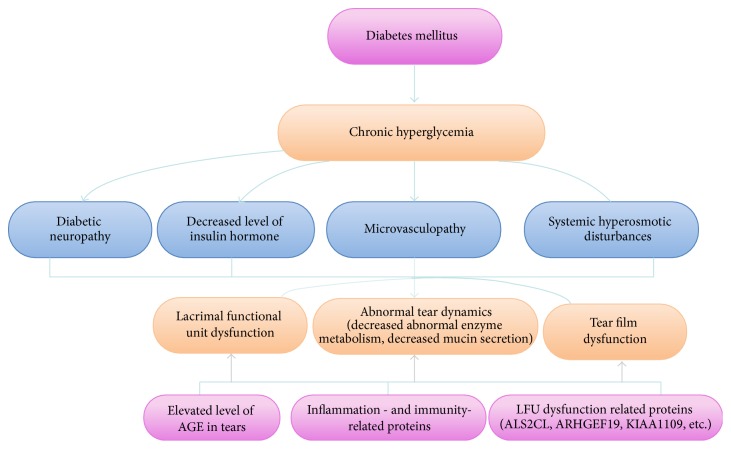
Etiology and pathogenesis of diabetes mellitus associated dry eye syndrome. Chronic hyperglycemia, diabetic periphery neuropathy, decreased insulin hormone, microvasculopathy, and systemic hyperosmotic disturbances are risk factors for diabetes-associated dry eye syndrome, which subsequently induce lacrimal function unit and tear film dysfunction and abnormal tear dynamics. Several proteins have been identified to be the contributor to diabetes mellitus associated dry eye syndrome.

Chronic hyperglycemia, diabetic periphery neuropathy, decreased insulin levels, microvasculopathy, and systemic hyperosmotic disturbances are risk factors for diabetes-associated DES (Figure 2).

Insulin is critical for proliferation of the acinar lacrimal gland (LG) and cornea epithelial cells. Insulin partially reversed the decreased protein expression induced by LG dysfunction; this process is involved in supporting exocytosis and vesicular formation through insulin replacement therapy [26]. It has been demonstrated that hyperglycemia induces histological alterations in the lacrimal gland, suggesting the role of diabetes-induced oxidative stress in DES [27]. Significant decreased reflex tearing was also reported in insulin dependent diabetic patients [23].

The glucose level is increased in the tears of diabetic patients [14]. A high glucose level in diabetic patients leads to elevated expression level of advanced glycation end-product- (AGE-) modified proteins. AGE-modified proteins in tears may be used as biomarkers to diagnose diabetes and/or DR [28].

Inflammation and immunity have been shown to play a prominent role in the pathogenesis of DES. Hyperglycemia initiates an inflammatory cascade that generates innate and adaptive immune responses of LFU. The downstream immune-inflammatory regulators have been identified to include matrix metalloproteinase-9 (MMP-9), immature antigen-presenting cells (APCs), CD4^+^ helper T cell (T_H_) subtype 1 and T_H_17 cell subsets, interferon (IFN) *γ* chemokines, chemokine receptors, cell adhesion molecules (CAMs), and interleukin-17 (IL-17) [29]. Furthermore, hyperglycemia causes tear film hyperosmolarity, inducing hyperosmolarity of the ocular surface epithelial cells, and stimulates a cascade of inflammatory events that involve MAP kinases and NFkB signaling pathways. The generation of inflammatory cytokines (e.g., interleukin-1A (IL-1A) and interleukin-1B (IL-1B), tumor necrosis factor-A (TNF-A), and matrix metalloproteinase-9 (MMP-9)) has also been demonstrated to be involved in the pathogenesis of DES [10, 30, 31].

Thousands of proteins have been identified and may be responsible for LFU dysfunction. Proteins (expressed in the human LFU) which are involved in the pathogenesis of diabetic DES include ALS2CL, ARHGEF19, KIAA1109, PLXNA1, POLG, WIPI1, ZMIZ2, and lacritin. The role of these proteins in patients with diabetic DES warrants further study [7]. The expression of apoptosis-related proteins, such as annexin A1, immunity- and inflammation-related proteins, including neutrophil elastase 2 and clusterin, and glycometabolism-related proteins, such as apolipoprotein A-II, has been reported to be increased in patients with DMDES [32].

## 6. Clinical Characteristics of Diabetes Mellitus Associated Dry Eye Syndrome

Diabetic patients with dry eye may have the same symptoms as DES without diabetes [2]. The symptoms consist of a gritty sensation, soreness, decreased visual acuity, photophobia, itching, decreased goblet cell density and corneal sensitivity, and tearing and pain concomitant with abnormalities in TUBUT, Schirmer's test, and corneal staining. More severe cases may be complicated by corneal lesions, conjunctivitis, keratopathy, and inflammation. It has been reported that gritty sensation is the most prominent symptom followed by the abnormalities of the tear film in patients with DMDES [2].

Dry eye symptoms are typically severe in patients with diabetes whose glycemic control is poor [33, 34]. Those with longer duration of diabetes may report fewer dry eye symptoms [16], and increased tear osmolarity is negatively correlated with symptoms. However, those without symptoms are unlikely to seek care. Lack of symptoms may result from a reduction in corneal sensitivity caused by diabetic peripheral corneal neuropathy [35]. Even a minimal decrease in corneal sensitivity is sufficient to cause changes in tear secretion. In a hospital-based study, longer duration of diabetes was associated with a lower (less severe) ocular surface disease index [2].

BUT (or NIBUT) and the Schirmer test are the most applied clinical methods used to diagnose DES. Tear osmolality and dynamics may also be used as supplementary diagnostic methods. In patients with diabetes, routine examination with BUT and Schirmer test is recommended. Early intervention is important to avoid visual impairment.

## 7. Prevention and Treatment Regimens of Diabetes Mellitus Associated Dry Eye Syndrome

Severe DMDES leads to visual impairment, corneal scarring, and ulcers, leading to secondary bacterial infections. The synergistic effect of corneal infection and diabetes accelerates corneal lesions, which irreversibly change the ocular surface and induce visual impairment [36]. Tear film dysfunction not only leads to the occurrence of dry eye but simultaneously aggravates the ocular surface, which induces a corneal epithelial defect, a common sign in diabetics [37].

The early diagnosis and treatment of dry eye are essential to avoid complications. The current treatment regimens for diabetic and nondiabetic dry eye patients are essentially the same. To date, there is no unified treatment option for DES. The application of artificial tears, including surfactants and various viscous agents, is predominately used to improve symptoms [38]. Artificial tears temporarily improve blurred vision and other symptoms. The drugs with anti-inflammatory effects do not comprise the active components such as growth factors which are contained in normal human tears [39, 40].

The most widely used anti-inflammatory drugs are corticosteroids, nonsteroidal anti-inflammatory drugs, cyclosporin A, tacrolimus, autologous blood serum, and several new drugs which are undergoing clinical trials [39, 41]. In patients with DMDES, corneal epithelial defects or side effects correlated with the topical drugs are more common than in those DES patients without DM; frequent routine follow-up for DMDES is necessary during treatment. Some devices are under development to help release the symptoms [42].

Topical corticosteroids reduce the signs, symptoms, and the level of inflammation in dry eyes and prevent corneal epithelial damage [43]. The ocular surface disease index score and dendritic cell density significantly improved by topical corticosteroids treatment [44]. The mechanisms of actions of corticosteroids on DES may be through suppression of cellular infiltration and increased synthesis of lipocortin which in turn block phosphorylation of phospholipase A2, which is the key step of the inflammatory cascade [41, 45]. However, side effects such as bacterial and fungal infections, increase in intraocular pressure, and cataracts have been reported [46]. Application of lower concentration of the steroids in short duration (one or two weeks) of the tropical steroid drug is recommended for those patients with DMDES.

To avoid side effects of topical steroids, nonsteroidal anti-inflammatory drugs (NSAIDs) are more commonly used instead of steroids in the clinic [47]. Pranoprofen, Bromfenac Sodium Hydrate, and RESTASIS® containing 0.05% cyclosporine have been applied in clinical practice. These topic drugs increase tear production, suppress immune response, and reduce damage to goblet cells induced by inflammation [48]. These drugs relieve the symptoms of aqueous-deficient dry eye and promote corneal epithelial recovery, but they do not improve tear production. Furthermore, these drugs reduce the sensitivity of the cornea, leading to corneal epithelium dissolution; they are recommended to be carefully applied to DM patients.

The mechanism of action of tacrolimus is similar to cyclosporin A, but the anti-inflammatory effect is stronger than that of cyclosporin A. It suppresses inflammation by inhibition of the expression of inflammation cytokines and chemokines [41, 49, 50].

Autologous blood serum eye drops have been shown to be effective on DES [51]. They contain immunoglobulins, vitamin A, fibronectin, growth factors, and anti-inflammatory cytokines which are the essential components present in natural tears. It has been found that 50% of the autologous serum eye drops are safe and effective for severe dry eye which is resistant to all other conventional treatments in a retrospective cohort study [52]. It has also been demonstrated that autologous serum tears are beneficial in the treatment of persistent corneal epithelial defect [53]. However, autologous serum tears do not have preservatives; they have a potential risk of inducing secondary infections; therefore, attention needs to be paid during the treatment, especially for those patients with DMDES.

Several drugs such as chemokine receptor antagonist, tofacitinib, LFA-1 antagonist, rebamipide (quinolinone derivative mucin secretagogue), MiM-D3 (nerve growth factor peptidomimetic, mucin secretagogue), EBI 005 (eleven biotherapeutics), diquafosol (P2Y2 receptor agonist), RU-101 (recombinant human serum albumin), KPI-121/LE-MMP 0.25%, and lifitegrast 5% (a small-molecule integrin antagonist) are undergoing clinical trials [41, 42]. Gene therapies that target LG have been demonstrated to be an alternative method in animal models of dry eye and specific treatment based on the pathogenesis of the condition in diabetic patients with dry eye warrants additional research [54].

In clinical practice, diabetics undergo regular fundus examinations. It has been suggested that the examination of the ocular surface and tear function also become part of the routine diabetic ophthalmic assessment and follow-up. Furthermore, preservative-free artificial tears and anti-inflammatory drugs are recommended to improve the hyperosmolar state of tears and to reduce the local inflammatory reaction. Protection of cornea and prevention of DMDES need to be considered in patients with islet dysfunction or poor glycemic control.

In summary, increasing prevalence of DMDES has been reported in recent years. In addition to the DR concerned, which is the leading cause of blindness, more attention should be paid to DMDES, the most frequent diabetic complication in eye disorders in clinical practice. The pathogenesis of diabetes-related DES remains elusive, and limited specific interventions are currently available. Additional clinical trials are warranted to confirm the effects of the currently applied drugs in diabetes-associated DES. Moreover, with the development of biomedical research, additional drugs, as well as gene and stem cell therapies, with specific targets will become available for the treatment of DES in diabetes.

## Abbreviations

AGE: Advanced glycation end-product
APCs: Antigen-presenting cells
BUT: Break-up time
CAMs: Cell adhesion molecules
DES: Dry eye syndrome
DM: Diabetes mellitus
DMDES: Diabetes mellitus associated dry eye syndrome
DR: Diabetic retinopathy
IFN: Interferon
IL: Interleukin
LFU: Lacrimal function unit
LG: Lacrimal gland
MMP-9: Matrix metalloproteinase-9
NPDR: Nonproliferative diabetic retinopathy
PDR: Proliferative diabetic retinopathy
PN: Polyneuropathy
TBUT: Tear break-up time test
TNF: Tumor necrosis factor.
